# Refractive corneal inlay for presbyopia in emmetropic patients in Asia: 6-month clinical outcomes

**DOI:** 10.1186/s12886-019-1069-2

**Published:** 2019-03-05

**Authors:** Gyule Han, Dong Hui Lim, Chan Min Yang, Gil Ho Park, Dae-Young Park, Hyun Seung Moon, Jae Myung Lee, Jong Ho Lee, Tae-Young Chung

**Affiliations:** 1 Department of Ophthalmology, Samsung Medical Center, Sungkyunkwan University School of Medicine, Seoul, South Korea; 2 Department of Preventive Medicine, Graduate School, The Catholic University of Korea, Seoul, South Korea; 3 Cheonan Kim’s Eye Clinic, Cheonan, South Korea; 4 Busan Balgeun Sesang Eye Clinic, Busan, South Korea; 5 Department of Ophthalmolgy, Jungang Hospital, Jeju, South Korea; 6 Gangnam First Eye Clinic, Seoul, South Korea; 7 Seoul Balgeun Sesang Eye Clinic, Seoul, South Korea; 8 Department of Ophthalmology, Samsung Medical Center, Sungkyunkwan University School of Medicine, #81 Irwon-ro, Gangnam-gu, Seoul, 06351 South Korea

**Keywords:** Presbyopia, Flexivue microlens, Corneal inlay

## Abstract

**Background:**

To evaluate the 6-month clinical outcomes of Flexivue Microlens refractive corneal inlay in emmetropic patients in Asia for the surgical compensation of presbyopia.

**Methods:**

In this retrospective study, corneal inlay implantation was done using a femtosecond laser. The follow-up period was 6 months. Near/intermediate/distant visual acuities, refraction, keratometry, defocus curve, wavefront aberrations, contrast sensitivity, Scheimpflug corneal scanning, endothelial cell density, dry eye test, confocal microscopy scanning, and patient questionnaires were evaluated.

**Results:**

The inlay implantation was performed in 21 eyes from June 2015 to April 2017. 6 months after surgery, the uncorrected near visual acuity of the operated eyes increased significantly from 0.55 ± 0.22 logMAR preoperatively to 0.25 ± 0.15 logMAR (*p* < 0.05) but mean bilateral uncorrected distant visual acuity did not change significantly (*p* = 0.90). Total higher-order aberration and spherical aberration increased, and the contrast sensitivity of the operated eyes decreased. Endothelial cell density and central corneal thickness did not change from preoperative values. Patient satisfaction for near vision was increased 6 months after implantation, and 50.0% of patients were independent of near spectacles. Explantation was done in 2 cases.

**Conclusion:**

The Flexivue Microlens refractive corneal inlay was effective for improving near visual acuity at 6 months follow-up But proportion of spectacle independency and patient satisfaction were lower in this Korean population than in previous reports. Further study with a longer follow-up period is needed.

## Background

The prevalence of presbyopia increases every year, and its effects on the quality of life for an aging population has placed presbyopia treatment in the forefront of research [1]. The therapeutic approaches to presbyopia cover the spectrum of lens procedures, from cataract surgery using multifocal, pseudo-accommodative intraocular lenses and monovision monofocal intraocular lenses [2–4] to corneal procedures such as laser-assisted in situ keratomileusis (LASIK) [5], conductive keratoplasty [6], and intracorneal lenses (corneal inlays).

Corneal inlays are perceived to be minimally invasive, easily reversible, and safe [7], so several types of inlays have been developed, including the KAMRA (AcuFocus Inc., Irvine, CA, USA), Raindrop Near Vision Inlay (ReVision Optics, CA), and Flexivue Microlens (Presbia Coöperatief U.A., Irvine, CA, USA). The KAMRA inlay increases the depth of focus using the pinhole principle [8]. Several studies have shown its effectiveness in increasing near visual acuity and its high patient satisfaction [8–10], and long-term follow-up studies reported stable results [11, 12]. The Raindrop Near Vision inlay changes the corneal curvature by creating a central hyperprolate cornea and enhances near vision by producing a region of increased corneal curvature in the center of the pupil [7]. Many clinical outcomes reported its efficacy for presbyopia correction [13, 14], including with a simultaneous LASIK procedure [15, 16]. Nonetheless, some surgeons have stopped implanting any intracorneal inlay for presbyopia because of wound healing concerns, unpredictable refractive outcomes, shifts in refractive errors, lens decentrations, and various optical side effects [17].

The Flexivue Microlens is a refractive optic corneal inlay that alters the corneal index of refraction to improve near vision performance by means of a bifocal optic that separates distance and near focal points [18]. Previous studies showed encouraging outcomes and safety, including increased near visual acuity, high patient satisfaction, and stable corneal structure [19–21]. But insufficient clinical evaluation of the Flexivue Microlens inlay has been done to confirm its efficacy and safety. Also, a racial factor may affect the results of corneal procedures [22], but no studies have yet reported results for the inlay in an Asian population. In this study, therefore, we analysed 6-month follow-up data on the efficacy and safety of the Flexivue Microlens inlay for the treatment of presbyopia in an Asian population.

## Methods

This is a retrospective study conducted at three separate institutions in Korea: the Department of Ophthalmology at Samsung Medical Center (Seoul, South Korea), the Seoul Balgeun Sesang Eye Clinic (Seoul, South Korea), and the Busan Balgeun Sesang Eye Clinic (Busan, South Korea) from June 2015 to April 2017. To clarify the efficacy and safety of the Flexivue Microlens inlay, we collected data on visual acuity changes (including the defocus curve test and contrast sensitivity test), patient satisfaction, dry eye testing, and structural changes after the implantation. This study followed the tenets of the Declaration of Helsinki, and permission was obtained from the Institutional Review Board of Samsung Medical Center.

The inclusion criteria were patients 45 to 65 years old with uncorrected near visual acuity (UNVA) worse than 20/50, uncorrected distance visual acuity (UDVA) better than 20/25, corrected distance visual acuity (CDVA) better than 20/20, a spherical equivalent (SE) of – 1.0D to + 1.0D, cylinder power ≤ 1.0D, central corneal thickness (CCT) ≥ 480 μm, and an endothelial cell count (ECC) ≥ 2000 per square millimeter (mm^2^) in the eye to receive the inlay. Exclusion criteria were a history of other ophthalmic diseases (retinal disease, glaucoma), intractable dry eye syndrome, corneal scar or opacity affecting visual function, keratoconus or irregular astigmatism, and inappropriate expectations about presbyopia correction.

### Corneal inlay

The Flexivue Microlens inlay is a transparent concave-convex disc, manufactured from a biocompatible hydrophilic acrylic material. Overall diameter is 3.2 and a thickness is approximately 15 to 20 μm, depending on the additional power. The central 1.6 mm diameter of the disc has no refractive power and the peripheral zone has the appropriate addition power. The available power ranges from + 1.50 D to + 3.50 D in 0.25 D increment. Central hole (0.51 mm diameter) permits transfer of oxygen and nutrients through the lens.

### Surgical techniques

All operations were performed by 2 surgeons (TYC and JHL). The inlay lens power was determined by lowering the near power refraction that the patient had tried and deemed comfortable for near work by + 0.25 D, according to the manufacturer’s instructions.

The surgical procedure was performed in the nondominant eye under topical anesthesia. The visual axis was marked with surgical pen based on the first Purkinje reflex. A pocket was created at a depth of 300 μm with a diameter of 5.5 mm, and the pocket tunnel was created with a width of 3.6 mm, and a length of 4.75 mm using a Femto LDV Z4 femtosecond laser (Ziemer, Port, Switzerland). Table 1 shows the femtosecond laser parameters. The incision side was determined at the higher astigmatism axis. Because all patients showed higher astigmatism at 180 degrees or no astigmatism, a temporal tunnel incision was created in all patients.

**Table 1:** Femtosecond laser parameters of the Flexivue Microlens inlay implantation

Femtosecond laser	Femto LDV Z4
Treatment type	Inlay
Tunnel width (mm)	3.60
Tunnel length (mm)	4.75
Tunnel depth (μm)	300
Tunnel spot separation (μm)	2
Tunnel line separation (μm)	2
Tunnel energy (nJ)	2
Pocket diameter (mm)	5.5
Side cut angle (°)	45

The inlay was loaded into the insertion device (Flexivue Microlens Inserter, Presbia Coöperatief U.A., Irvine, CA, USA). The instrument was inserted and then the inlay was released into the pocket, aligning its center hole with the central mark on the cornea.

The postoperative topical medication regimen was moxifloxacin 0.5% (Vigamox®) given 4 times daily for 2 weeks, prednisolone acetate 1% (Pred Forte®) given 3–4 times over 2 weeks and then converted to loteprednol etabonate 0.5% (Lotemax®) 3 times for 2 weeks, and then tapered over 2 months.

### Clinical examination

Ophthalmic examination was performed preoperatively and during postoperative visits (1 month, 3 months, and 6 months).

Distance visual acuity, near visual acuity (40 cm), intermediate visual acuity (80 cm), and manifest refraction were tested preoperatively and postoperatively. All tests were performed monocularly and binocularly. Ocular dominance was tested using the central hole test and a monovision trial.

A regular ophthalmic examination using a slit lamp ophthalmoscope was done at every visit. Dry eye was evaluated by tear-break-up time (BUT), Schirmer test, corneal staining score (Oxford score), and a standard Ocular Surface Disease Index (OSDI) questionnaire. A contrast sensitivity test was performed using the CSC-1000 instrument (Vector Vision, Greenville, Ohio) in photopic and mesopic conditions, binocularly and monocularly. Mesopic condition with glare (1 lx) contrast sensitivity was also tested after the test in the mesopic condition without glare. Keratometry and pachymetry were measured using Pentacam Scheimpflug tomography (Oculus, Wetzlar, Germany). Visante anterior segment optical coherence tomography (AS-OCT, Carl Zeiss, Oberkochen, Germany) was used to evaluate the pocket depth and post-implantation corneal changes. The corneal nerve fiber layer was scanned using a Confoscan 4 in vivo confocal microscope (Nidek, Gamagori, Japan), and then 1 measurer determined the subbasal nerve fiber density (NFD), nerve branch density (NBD), and maximal nerve fiber length (NFL) from the scanned images using ImageJ software (version 1.6.0_24, National Institutes of Health, Bethesda, USA). ECC was also measured by in vivo confocal microscopy (IVCM). A WASCA wavefront analyzer (Carl Zeiss Meditec, Jena, Germany) was used to measure total higher-order aberration (HoA), spherical aberration (SA), coma aberration, and trefoil aberration with a 5.0 mm diameter pupil. Defocus curves were plotted by measuring the visual acuity under photopic conditions at 5 m and adding lenses in + 0.5 D increments from – 4.0 D to + 2.0 D.

Corneal-inlay interface opacity was measured from cross-sectional Scheimpflug corneal images using caliper tools in the Pentacam Oculus software (scale from 0 = no clouding to 100 = tissue completely opaque). With line densitometry, we collected peak density values in the middle stroma (150 to 250 μm of corneal stroma) to eliminate a high density of endothelium and epithelium. Corneal density was obtained at 3 points: corneal apex and 1 mm from the corneal apex toward 3 o’clock and 9 o’clock considering an inlay diameter of 3.2 mm.

We evaluated patient satisfaction using a subjective questionnaire. Patients were asked to check the amount of their overall satisfaction for near, intermediate, and far vision (1 = very poor, 5 = excellent) and for their dependence on spectacles for near, intermediate, and distant work (always/sometimes/never). Symptoms of dysphotopsia (glare, halos, starburst, hazy vision and blurred vision) were assessed using the modified version from the Quality of Vision (QoV) questionnaire [23].

### Statistical analysis

Statistical analyses were performed using SPSS statistical software for Windows, version 21 (SPSS, Inc., Chicago, Illinois). Variables are expressed as the mean ± standard deviation, and a *p* value < 0.05 was considered statistically significant. We used paired *t* testing to compare preoperative and postoperative data. HoA, the contrast sensitivity test, and the QoV score were analyzed using the Wilcoxson-signed ranks test.

## Results

### Baseline characteristics and demographics

Twenty-one patients underwent inlay implantation from June 2015 to April 2017 (10 male, 11 female). The mean age was 52.7 ± 3.7 years (range 47–61 years). The operation was done on 13 right eyes and 8 left eyes. All 21 patients completed the 6 months of follow-up, and the mean follow-up period was 8.3 ± 4.9 months.

The preoperative average visual acuity was − 0.04 ± 0.07 (range − 0.18 – 0.00) logMAR bilateral UDVA, 0.35 ± 0.10 logMAR (range 0.20–0.52) bilateral UNVA, 0.14 ± 0.12 logMAR (range 0.00–0.30) bilateral uncorrected intermediated visual acuity (UIVA), − 0.01 ± 0.04 (range − 0.08 – 0.15) logMAR UDVA in operated eyes, 0.55 ± 0.22 (range 0.22–1.30) logMAR UNVA in operated eyes, and 0.26 ± 0.19 (range 0.00–0.80) logMAR UIVA in operated eyes. The mean ECC count was 2793 ± 312 cells per mm^2^, and the mean CCT was 540 ± 33 μm. The mean SE in the operated eyes was 0.20 ± 0.30D, and the mean refractive cylinder power was − 0.26 ± 0.36 D. The refractive power of the implanted inlay was 1.77 ± 0.25 D.

### Visual outcomes

Mean UNVA in the operated eyes increased significantly from 0.55 ± 0.21 to 0.26 ± 0.19 at 1 month, 0.25 ± 0.14 at 3 months, and 0.25 ± 0.15 logMAR at 6 months (paired T-test, *p* < 0.05). Bilateral UNVA also improved significantly from 0.35 ± 0.10 to 0.17 ± 0.11 logMAR (*p* < 0.05). The mean UIVA in the operated eyes changed from 0.26 ± 0.19 to 0.24 ± 0.12 logMAR, but that change was not significant (*p* = 0.64). Table 2 shows the visual acuity changes after implantation in detail.

**Table 2:** Pre- and postoperative near, intermediate, and distant visual acuity changes after flexivue microlens inlay implantation

		Preoperative	1 month	3 months	6 months
Operated eyes	UNVA	0.55 ± 0.22	0.26 ± 0.19*	0.25 ± 0.14*	0.25 ± 0.15*
	UIVA	0.26 ± 0.19	0.27 ± 0.17	0.27 ± 0.18	0.24 ± 0.13
	UDVA	0.01 ± 0.04	0.39 ± 0.21*	0.30 ± 0.18*	0.32 ± 0.14*
	CDVA	−0.02 ± 0.05	0.19 ± 0.13*	0.14 ± 0.12*	0.11 ± 0.10*
Bilateral	UNVA	0.35 ± 0.10	0.17 ± 0.11*	0.16 ± 0.08*	0.17 ± 0.11*
	UIVA	0.14 ± 0.12	0.09 ± 0.10	0.10 ± 0.11	0.07 ± 0.11
	UDVA	−0.04 ± 0.07	−0.03 ± 0.09	− 0.07 ± 0.08	−0.07 ± 0.11

*UNVA* uncorrected near visual acuity, *UIVA* uncorrected intermediate visual acuity, *UDCA* uncorrected distant visual acuity, *CDVA* best corrected distant visual acuity

*= significantly different versus preoperative value (*p* < 0.05, paired t-test)

SE changed from 0.20 ± 0.30 D preoperatively, to − 0.71 ± 0.72D 6 months postoperatively, showing a significant myopic shift after implantation (*p* < 0.05). The refractive cylinder power value 6 months after implantation was − 0.40 ± 0.39 D, which was not significantly different from the preoperative value, − 0.26 ± 0.36 D (*p* = 0.268).

CDVA and UDVA in the operated eyes decreased from − 0.02 ± 0.04 and 0.01 ± 0.04 to 0.19 ± 0.13 and 0.39 ± 0.21 at 1 month, 0.14 ± 0.12 and 0.30 ± 0.18 at 3 months, and 0.11 ± 0.10 and 0.32 ± 0.14 logMAR at 6 months (*p* < 0.05), respectively. 12 (60%) patients lost 1 line or more of CDVA, 6 (30%) patients lost 2 lines of CDVA in the operated eye, and no patient lost more than 3 lines. Bilateral UDVA did not change significantly (*p* = 0.90).

**Fig. 1:**
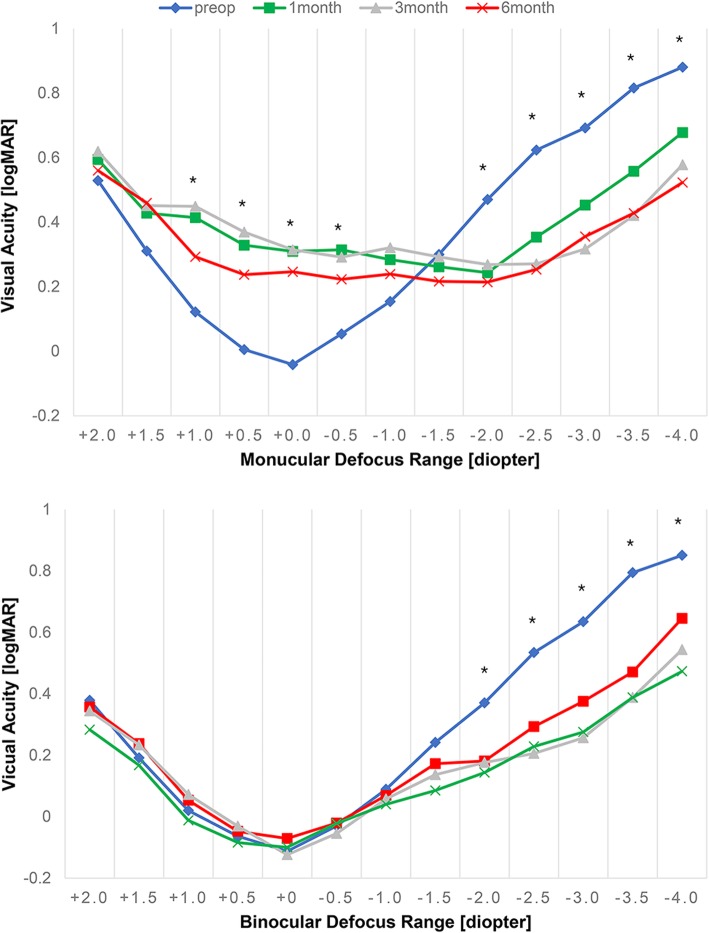
Defocus curve of Flexivue Microlens inlay. All values are represented as means. The X-axis is defocus (diopter), and the Y-axis is visual acuity (logMAR). * indicates significant differences between preoperative and 6-month values (*p* < 0.05)

The defocus curve test at 6 months showed significant improvement in visual acuity in the − 2.0 D to − 4.0 D range (*p* < 0.05). In the operated eyes, visual acuity in the + 2.0 D to − 0.5 D range decreased significantly, but in the bilateral test, no significant change was found in that range (Fig. 1).

### Corneal Keratometry, endothelial cell count, and Interface opacity

In topographical measurement, changes in corneal keratometry values for the anterior surface and posterior surface did not differ statistically preoperatively and 6 months postoperatively (*p* > 0.05). CCT at 6 months was 551 ± 30 μm, not significantly different from the preoperative value of 540 ± 33 μm (*p* = 0.16). Table 3 presents detailed changes in keratometric values and CCT.

**Table 3:** Keratometric values and central thickness of cornea after flexivue microlens inlay implantation

	Preoperative	1 month	6 months
Ant. steep K (D)	43.22 ± 1.21	43.24 ± 1.29	43.33 ± 1.10
Ant. flat K (D)	42.65 ± 1.15	42.25 ± 1.36*	42.40 ± 1.25
Post. steep K (D)	−6.42 ± 0.20	− 6.38 ± 0.17	− 6.34 ± 0.20
Post. flat K (D)	− 6.12 ± 0.16	− 6.06 ± 0.18	− 6.08 ± 0.20
Corneal thickness (μm)	540 ± 33	548 ± 30	551 ± 30

*D* Diopter, * significantly different versus preoperative value (*p* < 0.05, paired t-test)

6 months after implantation, the ECC was 2846 ± 351 per mm^2^, not significantly different from the preoperative count of 2793 ± 312 per mm^2^ (*p* = 0.42).

**Fig. 2:**
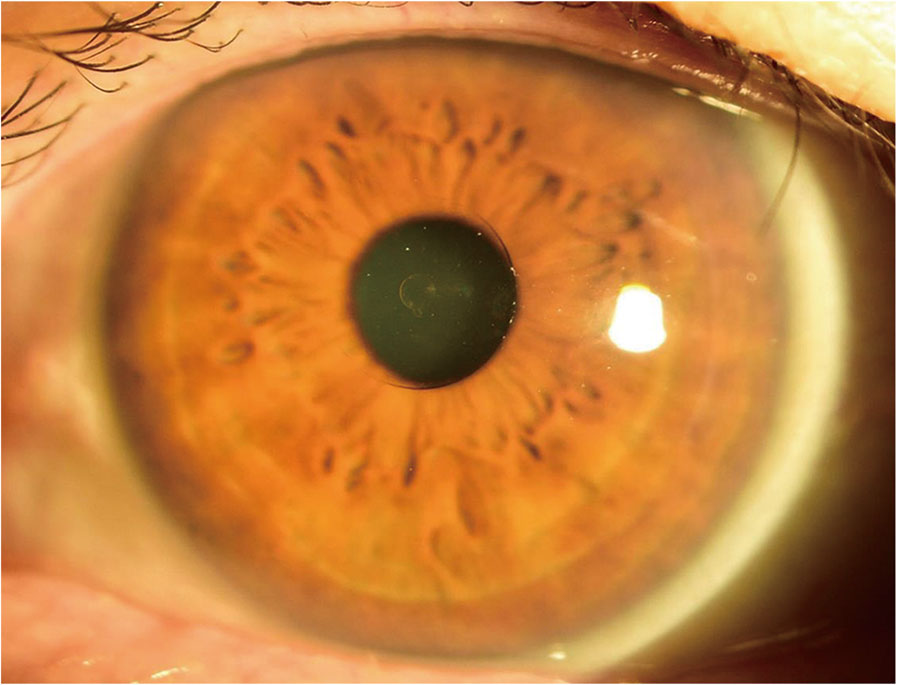
Cornea 6 months after Flexivue Microlens inlay implantation. Transparent inlay is seen at the center of the cornea. No sign of corneal thinning, inflammation, or vascularization is observed

Postoperative slit-lamp examinations showed clear corneas without signs of corneal thinning and well-centered inlays in all patients at every visit (Fig. 2). At 6 months, the average depth of the pocket, measure by AS-OCT, was 303 ± 29 μm (range 248 ~ 344 μm).

**Fig. 3:**
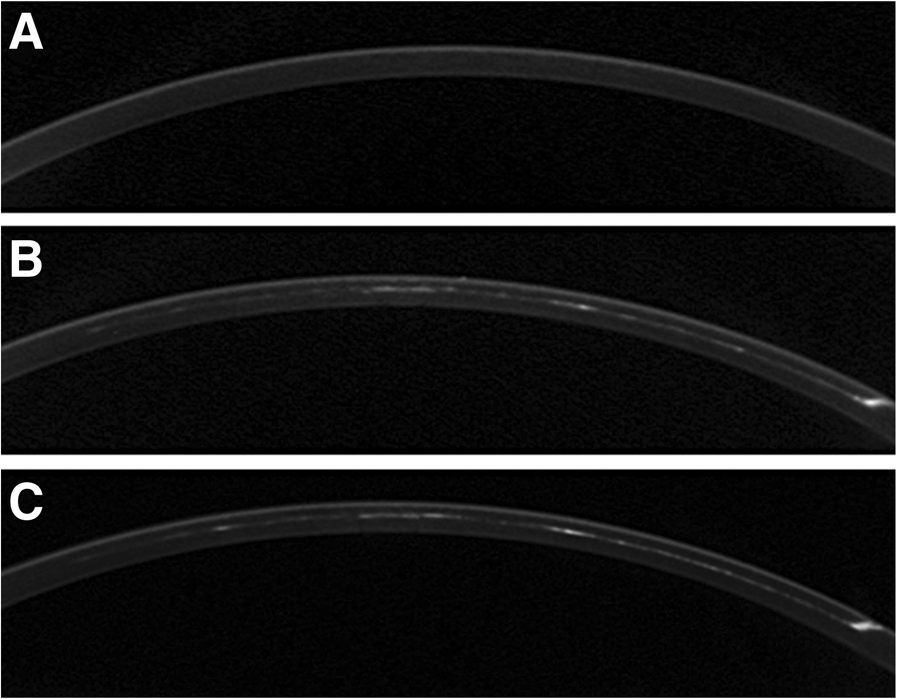
Cross-sectional Scheimpflug corneal images before and after Flexivue Microlens inlay implantation. Top: preoperative cornea. Middle: 1 month after implantation. Opacity is visible at the corneal-inlay interface and pocket tunnel area. Bottom: 6 months after implantation. The amount of opacity at the interface area is similar to that seen at 1 month

Measured baseline corneal density was 11.3 ± 2.4. The corneal-inlay interface density measured at 1 month and 6 months was 19.0 ± 5.2 and 16.8 ± 5.8, respectively, significantly greater than at baseline (*p* < 0.05), though the difference between 1 month and 6 months was not significant (*p* = 0.06, Fig. 3).

### Contrast sensitivity

**Fig. 4:**
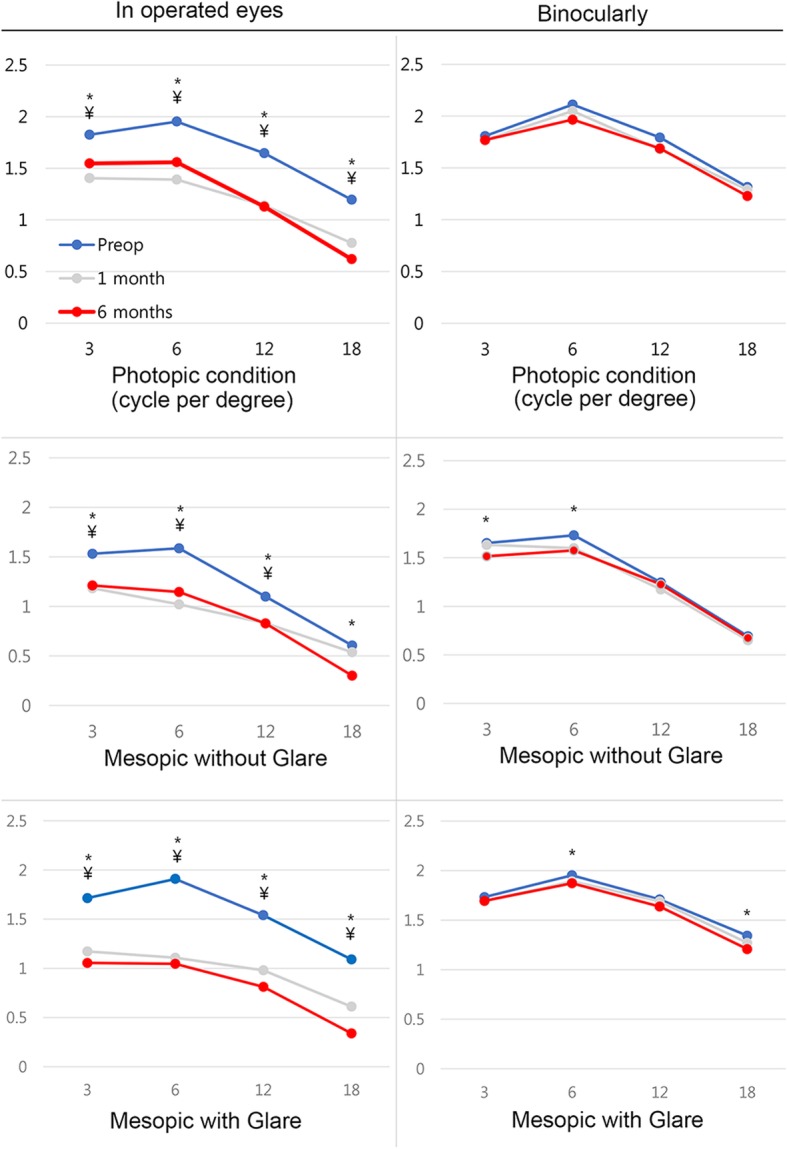
Contrast sensitivity test results before and after Flexivue Microlens inlay implantation. X-axis number represents the spatial frequency (cycle per degree). Significant differences (*p* < 0.05) between preoperative and 1-month values marked as ¥, and comparisons with 6-month values are marked as *

Contrast sensitivity at 1 month and 6 months decreased significantly in the operated eyes (*p* < 0.05) in both photopic and mesopic (with and without glare) environments at all frequencies. Binocular contrast sensitivity in the photopic condition showed no significant change at any frequency. But a significant decrease was observed at 3 cpd and 6 cpd in the mesopic condition without glare and at 6 cpd and 18 cpd in the mesopic condition with glare (*p* < 0.05). Figure 4 shows the change in contrast sensitivity after inlay implantation.

### Wavefront analysis

The root-mean-square (RMS) value of total HoA changed significantly from 0.25 ± 0.12 preoperatively to 0.32 ± 0.13 μm 6 months after implantation (*p* < 0.05). The Zernike value of SA also changed significantly from − 0.36 ± 0.13 μm preoperatively to 0.17 ± 0.18 μm 6 months postoperatively (*p* < 0.05). Changes in the RMS value of coma and trefoil aberration were not significant after implantation. Table 4 shows the mean pre- and postoperative aberration values in detail.

**Table 4:** Pre- and postoperative high-order aberration values for 5.0 mm pupils after flexivue microlens inlay implantation

	Preoperative	1 month	6 months
Total higher-order aberration (μm)	0.25 ± 0.12	0.32 ± 0.14*	0.32 ± 0.13*
Spherical aberration (μm)	−0.36 ± 0.13	0.16 ± 0.17*	0.17 ± 0.18*
Coma (μm)	0.29 ± 0.16	0.31 ± 0.14	0.30 ± 0.19
Trefoil (μm)	0.27 ± 0.24	0.41 ± 0.34	0.37 ± 0.28

Total higher-order aberration, coma, and trefoil are presented as root-mean-squared values. Spherical aberration is presented using the Zernike value. * = significantly different versus preoperative value (*p* < 0.05, Wilcoxson-signed ranks test)

### Corneal nerve Fiber analysis and dry eye evaluation

**Fig. 5:**
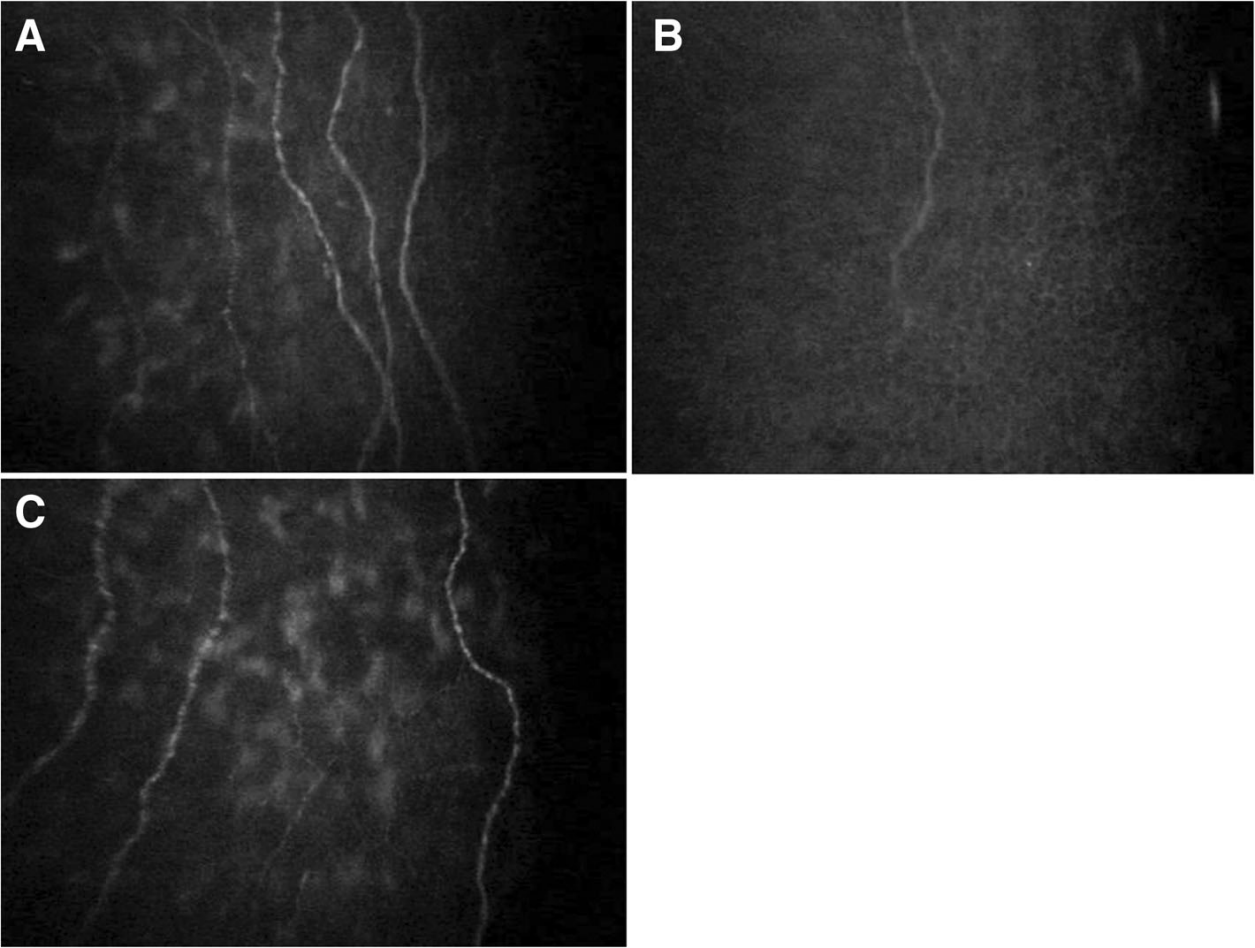
Confocal microscopic photographs of cornea before and after Flexivue Microlens inlay implantation. *A*: Before implantation. A few corneal nerve fibers are observed. *B*: Postoperative 1 month. The number of nerve fibers has decreased. *C*: Postoperative 6 months. Nerve fibers have regenerated, and the fiber density is similar to the preoperative state

The average corneal NFD, which was 18.1 ± 10.5/mm2 preoperatively, significantly decreased to 10.6 ± 5.0/mm^2^ at 1 month (*p* < 0.05) and recovered to 15.4 ± 7.6/mm^2^ at 6 months (*p* = 0.17, comparing to preoperative value). The NFL and NBD at 1 month were 3.2 ± 2.5 and 5.2 ± 3.6, respectively, also significantly decreased from the preoperative values of 6.0 ± 3.3 and 18.5 ± 12.3, but they recovered to 5.2 ± 3.6 and 14.6 ± 12.1, not significantly different from preoperative values, at 6 months (*p* = 0.32, 0.22, respectively, Fig. 5).

BUT decreased significantly at 1 month and 3 months (*p* = 0.03, 0.01, respectively) but showed no significant difference from the preoperative value at 6 months (*p* = 0.636). The corneal staining score and OSDI at 1 month had decreased significantly, but they recovered at 3 months and 6 months. Postoperative Schirmer testing showed no significant differences from preoperative values (Table 5).

**Table 5:** Dry eye values after flexivue microlens inlay implantation

	Preoperative	1 month	3 months	6 months
BUT (sec)	4.18 ± 1.42	2.81 ± 1.22*	3.75 ± 1.00*	3.43 ± 1.31
Schirmer test	7.31 ± 4.08	6.00 ± 4.41	6.13 ± 4.79	6.43 ± 3.61
Oxford score	0.38 ± 0.72	1.00 ± 1.46*	0.25 ± 0.58	0.31 ± 0.48
OSDI	23.1 ± 15.2	36.67 ± 15.6*	25.8.1 ± 114.5	24.9 ± 13.0

*BUT* Tear break-up time, *OSDI* Ocular Surface Disease Index, * **=** significantly different versus preoperative value (*p* < 0.05, paired t-test)

### Subjective patient questionnaire

**Fig. 6:**
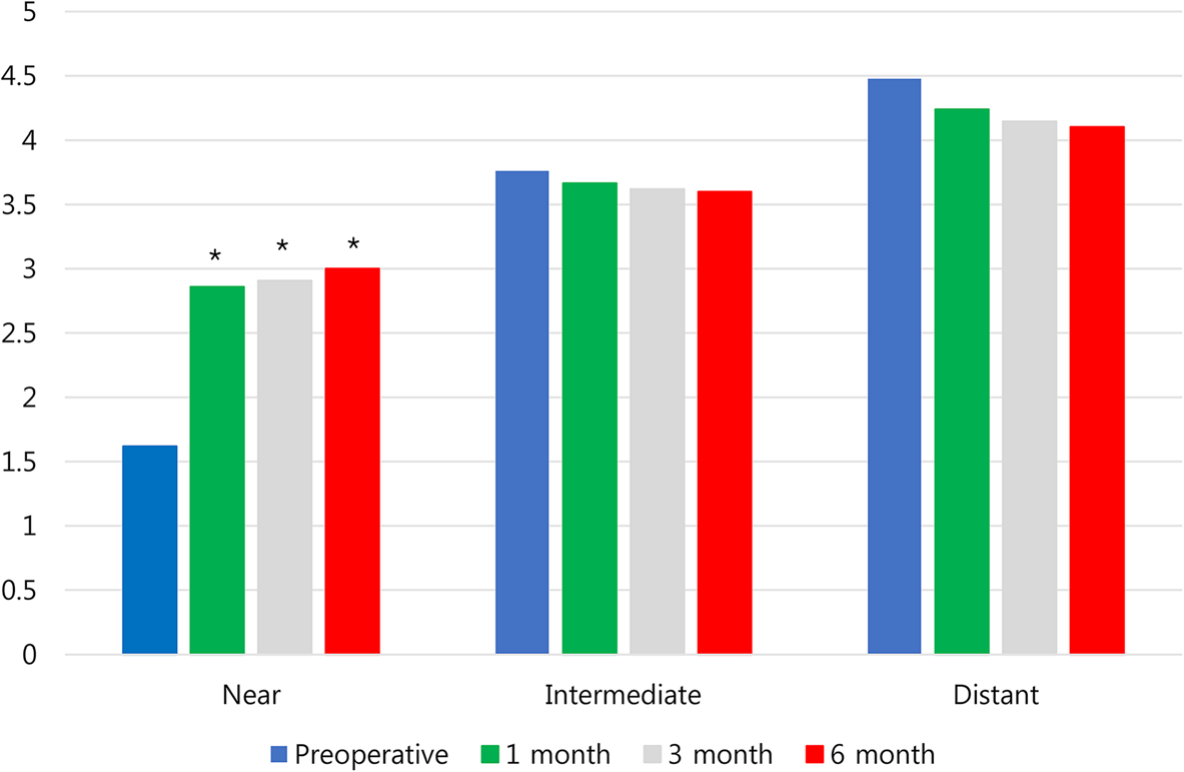
Satisfaction score for near, intermediate, and distant vision after Flexivue Microlens inlay implantation. * indicates significant differences from preoperative values (*p* < 0.05)

**Fig. 7:**
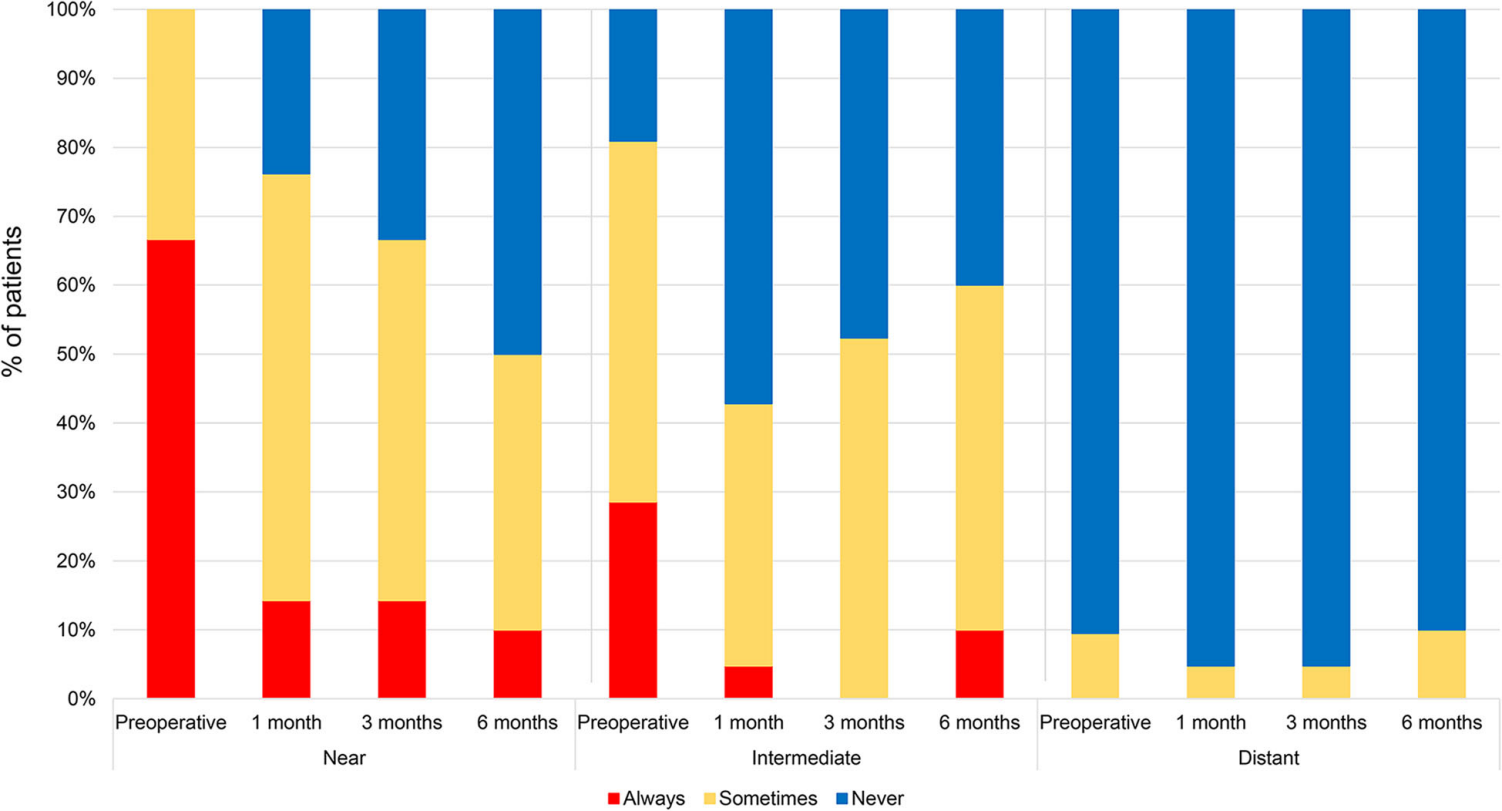
Changes in the proportion of patients using near, intermediate, and distance spectacles before and after Flexivue Microlens inlay implantation

At 6 months after inlay implantation, the mean satisfaction score for near vision was 3.00 ± 0.73, a significant improvement from the preoperative score of 1.62 ± 0.50 (*p* < 0.05, Fig. 6). 50% of patients answered that they never used near spectacles for near tasks. For intermediate vision, 40% were independent of spectacles. No patient used spectacles for their distant vision (Fig. 7).

**Fig. 8:**
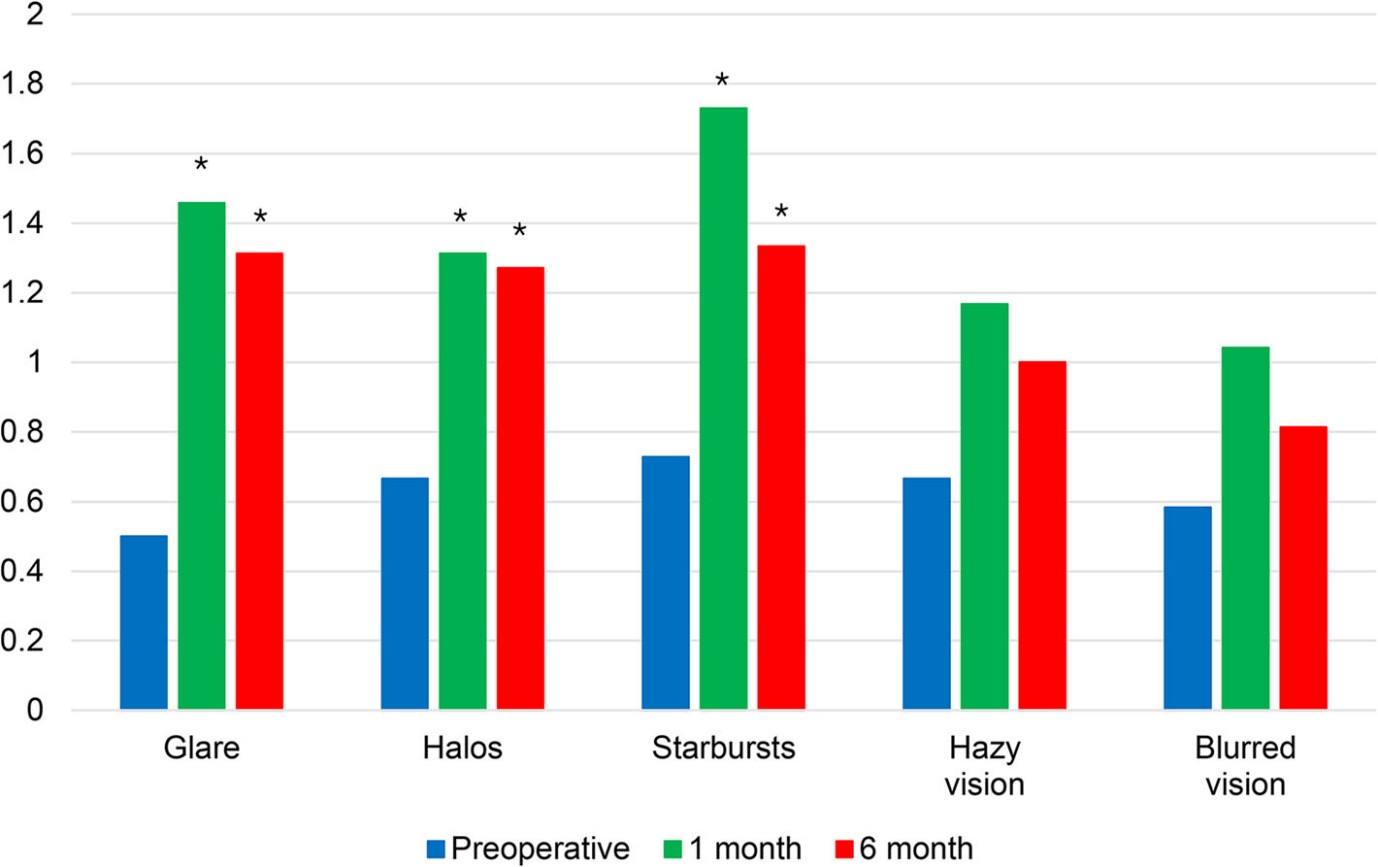
Dysphotopsia scores (from modified QoV questionnaire) after Flexivue Microlens inlay implantation. * indicates significant differences compared to preoperative values (*p* < 0.05)

Average score for glare, halo and starburst increased significantly 6 months postoperatively compared with preoperative scores; otherwise hazy vision and blurred vision showed no significant change (Fig. 8).

### Safety and complications

Explantation was done in 2 cases (9.5%). In one case, the inlay was removed after 3 months follow-up; the other removal was done after 6 months follow-up. The patients had complaints about blurred vision and glare, and UDVA was 20/40. After removal of the inlay, UDVA recovered to 20/20, and the blurred vision disappeared 1 week after removal.

## Discussion

Several previous studies considered Flexivue Microlens inlay implantation. Limnopoulou et al. [19] reported the results of 47 patients 1 year after Flexivue Microlens implantation. They found that the mean bilateral UNVA improved significantly to 0.13 logMAR, and the mean UDVA of the operated eyes decreased to 0.38 logMAR, though binocularly it did not change significantly. They also reported that 37% of patients lost 1 line of CDVA in the operated eye, with a significant decrease to 0.10 logMAR. The mean SE refraction of the operated eyes showed a significant myopic shift from 0.66D to − 1.95D, and they did not report any inlay removal. In a study of the 3-year results of 86 patients who underwent Flexivue Microlens implantation, Maladrini et al. [20] reported that 26 patients showed a mean preoperative UNVA and UDVA of 0.76 logMAR and 0.00 logMAR, respectively, compared with postoperative UNVA and UDVA of 0.10 logMAR and 0.15 logMAR, respectively. The mean UDVA, both in the treated eyes and binocularly, worsened at 12 months and then remained stable until 36 months. They found that 8% of patients lost more than 1 line of CDVA, but no patient lost more than 2 lines. The mean refraction shifted slightly at 12 months, 24 months, and 36 months, but those shifts were not significant. Explantation was performed in 6 treated eyes (7.4%) because of halos, glare, and reduced UDVA. Beer et al. [21] reported the 1-year clinical outcomes of 31 patients. The mean UNVA improved to 0.17 logMAR, and 87.1% of the patients showed 0.3 or better UNVA. 83.9% of the patients lost 1 line in CDVA in the operated eye, and 16.1% lost more than 3 lines. The mean SE refractive error decreased significantly from 0.33D preoperatively to − 1.78D.

We found that the UDVA of the operated eyes decreased significantly after implantation, though changes in binocular UDVA were not significant. The mean UNVA in the operated eyes improved significantly from 0.55 logMAR preoperatively to 0.25 logMAR at 6 months. That postoperative UNVA improvement was smaller than found in the previous studies. UIVA was not affected by inlay implantation. 30% of patients in our study lost 2 lines of CDVA in the operated eye (60% lost 1 line or more), unlike the patients in the first two studies. Given that our follow-up period was shorter than in the other studies, additional improvement can be expected. We found that corneal-inlay interface opacity was maintained after 6 months, which could explain the lowered near and distant visual acuity. In corneal morphologic research for the Flexivue Microlens [24], an early-detected hyperreflective area in the inlay surface and borders contained activated hyperreflective keratocyte and extracellular matrix with cellular debris from the degeneration of keratocytes, and that activity decreased with time, though it seems to have lasted for 6 months in our results. A previous study reported that the incidence of corneal haze after photorefractive keratectomy was significantly higher among patients with brown irides than among patients with blue irides [22], and a study performed in Korea noted that corneal haze after surface ablation is much more common than reported previously [25]. Those findings imply that corneal wound healing might be affected by racial differences and be more aggressive among Asian people. Also, there might be racial differences to the foreign body reaction against corneal inlays that could have affected our clinical results. Among our patients, explantation was done in 2 cases (9.5%). Observation for 6 months is insufficient to conclude the level of wound healing and we expect this opacity will be decreased at further follow-up, with better distant visual acuity.

Contrast sensitivity test results vary among the previous studies [19–21]. In our study, contrast sensitivity decreased in all frequencies under photopic conditions and mesopic conditions with glare and in low frequencies under the mesopic condition without glare. Many scientific studies have shown that contrast sensitivity is a robust indicator of functional vision [26–28], and the loss of contrast sensitivity after femtosecond laser procedures is considered to be a factor that worsens vision quality [29]; therefore, the decrease in contrast sensitivity after Flexivue Microlens implantation might play a role in lowering patient satisfaction and worsening UNVA.

A significant myopic shift occurred after implantation, but the amount of change (from 0.20D to − 0.71D) was smaller than the mean power of the implanted inlays (1.77D). Measuring the manifest refraction after the inlay implantation can be difficult because of irregular reflex originated from the regional difference of refractive power over cornea. This discrepancy could be the result from refraction inaccuracy. But in previous study also, Maladrini [20] reported that the refractive power change was not significant between the preoperative value and 3 years postoperatively. Only inaccuracy is not the enough factor for the discrepancy and the exact explanation cannot be found in this study, but we could assume that the refractive power change through the corneal inlay might not be the only action of presbyopic correction. Extended depth of focus from the induced aberration seems to be the part of the correction mechanism as discussed below.

In a previous study, the RMS of HoA and SA changed significantly after the procedure [19–21]. We also observed change in SA and a postoperative increase in HoA. Because we present SA values using Zernike values, the direction of change must be interpreted differently from the values given in the previous study [20], but this change also implies an extended depth of focus. Charman [30] stated that the main requirement in presbyopia is an extended depth of focus to ensure adequate near and distance vision with good retinal contrast, rather than achieving the highest level of acuity and modulation transfer function at a single distance. Limnopoulou [19] noted that increased aberration after Flexivue Microlens implantation could positively affect near vision by increasing ocular depth of focus, even though it could also influence distance visual performance. This induced aberration can be attributed to the decrease in contrast sensitivity at high spatial frequencies [31, 32], but it could also play a role in increasing the depth of focus to acquire adequate near and distance vision. We consider both refractive power correction and increasing the depth of focus to be important effects of the inlay in compensating for presbyopia.

The defocus curve of the inlay in our study shows that patients had significantly better vision in the near range without deteriorating distance vision bilaterally, indicating the efficacy of the Flexivue Microlens inlay for improving near vision intuitively by visual format.

Corneal keratometry values on the anterior and posterior surface did not change from their preoperative values after implantation, and the mean CCT remained stable throughout the follow-up period. Those results suggest that minimal change occurred in the corneal structure within the first 6 months of inlay implantation. Considering the inlay’s structure (relatively thicker peripheral zone than central hole and plano central zone), CCT might not be the exact representative value of corneal structural change after the inlay implantation. However, the inlay diameter (3.2 mm) and central zone (1.6 mm) are small, CCT can be the acceptable representative value. No sign of corneal thinning or melting was observed. ECC also stayed the same. Thus, the corneal structure after the inlay implantation was stable until 6 months after the operation but longer follow-up results are needed. The regular follow-up has been done until recent days (up to 2 years) and we haven’t experienced the safety problem.

According to the AS-OCT and IVCM observations by Malandrini et al. [24] and the IVCM scanning results from Limnopoulou et al. [19], 1 year after implantation, the inlay elicited a low-level wound-healing response in its immediate vicinity with no alteration in the corneal structure. However, in this study, corneal-interface opacity remained 6 months after implantation. Thus, the wound-healing response might last longer than was previous reported.

Corneal subbasal NFD, NFL, and NBD, measured from IVCM scans, all decreased at 1 month and then recovered to their preoperative levels by 6 months. Corneal procedures such as LASIK decrease the subbasal NFD, and the regeneration of subbasal nerve fiber was reported to be slow [33]. Corneal inlay implantation carries the same risk of corneal nerve fiber loss. But as revealed in our results, regeneration to the preoperative state was relatively rapid. In a study comparing nerve-fiber regeneration after small incision lenticule extraction (SMILE) and femtosecond laser–assisted LASIK [34], early nerve fiber regeneration occurred faster in eyes receiving SMILE. The author noted that early regeneration in the SMILE group was likely related to the shorter side cut and smaller diameter of the lamellar cut compared with the LASIK group. We assume that because corneal inlay implantation requires an even shorter side cut and smaller lamellar cut then SMILE, faster nerve regeneration is possible, allowing the nerve fiber state to reach its preoperative level within 6 months.

Many studies have reported a strong relationship between corneal nerve denervation and dry eye [35, 36]. We found that the BUT, Oxford score, and OSDI worsened at 1 month postoperatively and then recovered at 6 months, showing the same tendency as corneal nerve fiber. Intensive care for dry eye might be necessary at the early postoperative stage.

Patient satisfaction levels for near vision increased significantly after implantation without changing the satisfaction levels for intermediate or distant vision. 50.0% of patients answered that they never used spectacles for near work, whereas Limnopoulou et al. [19] reported that 81.25% of their patients perceived their UNVA as excellent, and 93.75% were independent of their spectacles. Also, Beer et al. [21] reported that 91% of their patients subjectively rated their UNVA as excellent or good, and 65% never used spectacles for near vision. Direct comparisons to previous studies might be irrelevant because of different scoring systems, but our study seems to report relatively low patient satisfaction. A study of the KAMRA inlay performed in Korea [37] also appeared to show lower overall satisfaction than similar reports from other places. Thus, the results of the inlay for presbyopia might vary among races. Lower UNVA after implantation might be one reason for that racial difference, and although it might be inaccurate for the inlay patient due to the scissoring reflex, the actual refractive power of the inlay might be weaker than the anticipated level, which would lower the UNVA and independency from near spectacles. Yoo et al. [38] noted that the lower proportion of near spectacle independency after inlay implantation might indicate a cultural difference: Koreans require high visual resolution to read Korean characters, most of which have more strokes than Roman characters; therefore closer distances for reading are required for Korean texts than for Western ones. In addition, because Asian people typically have shorter arms than Caucasians [39], the near-work distance at which people feel comfortable might differ. Therefore, because Asian people tend to read books and use a cellphone closer than Western people, the suggested refractive power for the inlay for Western people might be too weak for Asian patients. We conducted 2 operations not included in this study in which we used a higher power (by + 0.25 D – + 0.50 D) for the implanted inlay than the company recommends, and those patients reported good satisfaction, although those results are preliminary.

The limitations of the current study were the small number of patients and short follow-up duration. A large number of patients with longer observation is needed to ensure the efficacy and long-term safety of inlay implantation. Regarding the safety issues of the corneal inlay have been occurred recently, longer follow-up study is essential for the conclusion of safety. Also epithelial and stromal thickness analysis could be very useful for the interpretation of the factors for relatively lower UNVA but was not included in this study.

## Conclusions

At 6 months postoperatively, the Flexivue Microlens refractive corneal inlay was effective for improving near visual acuity. However, satisfaction with near vision and proportion of spectacle independency were lower than in previous studies.

## Abbreviations

AS-OCT: Anterior segment optical coherence tomography
BUT: Tear-break-up time
CCT: Central corneal thickness
CDVA: Corrected distant visual acuity
cpd: Cycle per degree
ECC: Endothelial cell count
HoA: Higher-order aberration
IVCM: In vivo confocal microscopy
LASIK: Laser-assisted in situ keratomileusis
NBD: Nerve branch density
NFD: Subbasal nerve fiber density
NFL: Maximal nerve fiber length
OSDI: Ocular Surface Disease Index
RMS: Root-mean-square
SA: Spherical aberration
SE: Spherical equivalent
UDVA: Uncorrected distance visual acuity
UIVA: Uncorrected intermediated visual acuity
UNVA: Uncorrected near visual acuity
